# BGATT-GR: accurate identification of glucocorticoid receptor antagonists based on data augmentation combined with BiGRU-attention

**DOI:** 10.1038/s41598-025-05839-8

**Published:** 2025-07-01

**Authors:** Watshara Shoombuatong, Pakpoom Mookdarsanit, Nalini Schaduangrat, Lawankorn Mookdarsanit

**Affiliations:** 1 Center for Research Innovation and Biomedical Informatics, Faculty of Medical Technology, Mahidol University, Bangkok, 10700 Thailand; 2 Computer Science and Artificial Intelligence, Faculty of Science, Chandrakasem Rajabhat University, Bangkok, 10900 Thailand; 3 Business Information System, Faculty of Management Science, Chandrakasem Rajabhat University, Bangkok, 10900 Thailand

**Keywords:** Glucocorticoid receptor, QSAR, Data augmentation, Machine learning, Self-attention, Bidirectional gated recurrent unit, Computational biology and bioinformatics, Data mining, Machine learning

## Abstract

The glucocorticoid receptor (GR) is a critical nuclear receptor that regulates a broad spectrum of physiological functions, including stress adaptation, immune response, and metabolism. Given the association between aberrant GR signaling and various pathological conditions, this pathway represents a promising therapeutic target. Several GR antagonists have been developed to block glucocorticoid binding to the receptor, showing therapeutic potential in disorders characterized by heightened or dysregulated glucocorticoid signaling. Therefore, this study proposes an innovative deep learning-based hybrid framework (termed BGATT-GR) that leverages a data augmentation method, a bidirectional gated recurrent unit (BiGRU), and a self-attention mechanism (ATT) to attain more accurate identification of GR antagonists. In BGATT-GR, we first employed AP2D, CDKExt, KR, Morgan, and RDKIT to extract molecular descriptors of GR antagonists and combined these molecular descriptors to generate multi-view features. Second, we adopted a data augmentation method that combined both random under-sampling (RUS) and the synthetic minority over-sampling technique (SMOTE) to address the issue of class imbalance. Third, the BGATT architecture was constructed to enhance the utility of the multi-view features by generating informative feature embeddings. Finally, we applied principal component analysis (PCA) to reduce the dimensionality of these feature embeddings and fed the processed feature vectors into the final classifier. Extensive experimental results showed that BGATT-GR provided more stable performance in both cross-validation and independent tests. Furthermore, the independent test results revealed that BGATT-GR attained superior predictive performance compared with several conventional ML models, with a balanced accuracy of 0.957, an MCC of 0.853, and an AUPR of 0.962. In summary, our experimental results provide strong evidence to suggest that BGATT-GR is highly accurate and effective for identifying GR antagonists.

**Supplementary Information:**

The online version contains supplementary material available at 10.1038/s41598-025-05839-8.

## Introduction

The glucocorticoid receptor (GR) is a critical nuclear receptor that regulates a broad spectrum of physiological functions, including stress adaptation, immune response, and metabolism^1,2^. Upon binding of glucocorticoid ligands, the glucocorticoid receptor (GR) undergoes nuclear translocation, where it regulates gene transcription via interactions with glucocorticoid response elements^3^. Given the association between aberrant GR signaling and various pathological conditions, this pathway represents a promising therapeutic target. Several GR antagonists have been developed to block glucocorticoid binding to the receptor, showing therapeutic potential in disorders characterized by heightened or dysregulated glucocorticoid signaling.

One of the most established applications of GR antagonists is in Cushing’s syndrome, a condition characterized by chronic hypercortisolism. Excess glucocorticoid activity in Cushing’s syndrome results in severe metabolic disturbances, including hyperglycemia, obesity, and cardiovascular complications^4,5^. GR antagonists, such as mifepristone, have demonstrated efficacy in improving glycemic control and reducing other systemic effects of the disease^6–8^. Furthermore, glucocorticoid levels elevated in response to fasting or stress and leading to obesity can be modulated by GR antagonists providing a promising approach for the treatment of metabolic disorders^9^. In psychiatry, GR antagonists have demonstrated potential for treating major depressive disorder, particularly in patients with dysregulated hypothalamic-pituitary-adrenal axis function and elevated cortisol levels. By modulating GR activity, these antagonists can reduce the physiological effects of hypercortisolemia, alleviating depressive symptoms in treatment-resistant populations^2,10^. In oncology, GR antagonists are being investigated in prostate cancer, where GR signaling can bypass androgen receptor (AR) inhibition, contributing to therapy resistance. Preclinical studies suggest that GR antagonism may restore sensitivity to AR-targeted therapies, offering a potential therapeutic avenue for castration-resistant prostate cancer^11,12^.

The discovery of novel GR antagonists has been significantly advanced by machine learning (ML) and deep learning (DL), which offers powerful tools for identifying and optimizing potential drug candidates. For instance, recent studies have employed ML-based models to predict the activity of small molecules against GR, enabling rapid screening of chemical libraries^7,13–15^. Furthermore, ML techniques have been integrated into the discovery of anti-obesity compounds with GR antagonist activity, leveraging structural and pharmacokinetic data to refine candidate selection^16^. These advancements illustrate the potential of computational approaches to accelerate drug discovery while reducing costs and experimental workload underscoring the synergy between computational and experimental methods in the development of GR-targeted therapies, paving the way for more effective and precise treatments for GR-related diseases.

Here, we introduce BGATT-GR, a novel DL-based hybrid framework designed for the rapid and precise identification of novel GR antagonists from large-scale uncharacterized compounds. The contributions of this framework are summarized in the following three aspects:

(i) To address the imbalanced dataset, BGATT-GR employed a data augmentation method that utilized both random under-sampling (RUS) and synthetic minority over-sampling technique (SMOTE) to construct balanced datasets. To the best of our knowledge, this is the first instance of combining SMOTE and RUS strategies for the identification of GR antagonists.
(ii) To improve the discriminative power of the features, we constructed the BGATT architecture, which combines a bidirectional gated recurrent unit (BiGRU) with a self-attention mechanism (ATT) to capture the sequential and semantic information of GR antagonists. Specifically, we used five well-known molecular descriptors, including AP2D, CDKExt, KR, Morgan, and RDKIT, to represent GR antagonists and combined these molecular descriptors to generate multi-view features. The multi-view features were then fed into the BGATT architecture to generate crucial feature embeddings.
(iii) Extensive experimental results demonstrated that BGATT-GR can precisely identify GR antagonists, significantly outperforming several conventional ML classifiers, such as multilayer perceptron (MLP), AdaBoost (ADA), random forest (RF), extreme gradient boosting (XGB), and support vector machine (SVM). BGATT-GR achieved a balanced accuracy (BACC) of 0.957, a Matthews correlation coefficient (MCC) of 0.853, and an area under the precision-recall (PR) curve (AUPR) of 0.962.

## Materials and methods

### Data preparation

For this study, small molecule compounds targeting the glucocorticoid receptor (Target ID: CHEMBL2034) were sourced from the ChEMBL database^17^. Initially, 13,227 compounds demonstrating activity against GR were retrieved. Data curation was then performed using custom in-house scripts developed in the R programming environment^18^. During this curation phase, only compounds marked with an equal symbol corresponding to the bioactivity column were retained, while those annotated with any other symbols were discarded. Redundant entries and missing data points were also excluded. The dataset was further refined by filtering for compounds with bioactivity measured as IC_50_ and maintaining a standard deviation of 2. This refinement resulted in a dataset comprising 1,632 compounds. To improve interpretability and enable consistent comparisons at identical molar levels, IC_50_ values were transformed into their pIC_50_ equivalents. Compounds were then characterized based on their pIC_50_ values: those with pIC_50_ greater than 6 were classified as active (referred as positive class), while those with values below 5 were deemed inactive (referred as negative class). Compounds with pIC_50_ values ranging from 5 to 6 were labeled as intermediate and excluded from further analysis. More detailed procedure regarding the curation phase is in line with our previous studies^19–22^. Ultimately, the refined dataset comprised 1,314 active compounds and 275 inactive compounds. Among these, 263 active compounds and 55 inactive compounds were randomly selected as the independent test dataset, while the remaining compounds were used to construct training dataset.

### Molecular descriptor extraction

Molecular feature extraction entails the characterization of compounds using both quantitative and qualitative descriptors, which capture their structural attributes, physicochemical properties, and connectivity^28^. In this study, preprocessing was conducted using the PADEL-descriptor software^23^, encompassing steps such as salt removal, duplicate elimination, and tautomer standardization. Following these preprocessing procedures, molecular descriptors were generated using the SMILES representations of the analyzed compounds. Five unique molecular descriptors types were employed in the study, namely AP2D, CDKExt, KR, Morgan, and RDKIT^24–28^. These molecular descriptors have been widely used in several successful applications in computational chemistry^29–34^. The calculations associated with generating these molecular descriptors were executed using both Python and R programming environments^18,35^. Detailed descriptions of each fingerprint type can be found in Table 1.

**Table 1:** Summary of five SMILES-based feature descriptors used in this study.

Fingerprint	#Feature	Description	Ref
AP2D	780	Presence of atom pairs at various topological distances	^28^
CKDExt	1,024	Extends the fingerprint with additional bits describing ring features	^24^
MACCS	166	Binary representation of chemical features defined by MACCS keys	^27^
Pubchem	881	Binary representation of substructures defined by PubChem	^26^
FP4C	307	Count of SMARTS patterns for functional groups	^25^

### Data augmentation method

In this study, the ratio between the numbers of active and inactive compounds in the training dataset is approximately 1:5. A highly imbalanced dataset can lead to a bias toward the majority class samples (active compounds)^36,37^, resulting in a high number of false positives. To mitigate this issue, we applied a designed data augmentation method that combines both RUS and SMOTE. Specifically, the SMOTE method was employed at different proportions, including 0%, 25%, 50%, 75%, and 100%, to oversample the negative samples. Based on the same proportions applied to the negative samples, the RUS method was used to randomly under-sample the positive samples, thereby generating balanced training datasets with a ratio of 1:1 between active and inactive compounds^38^. Supplementary Table**S1** provides a statistical summary of the training and independent test datasets across the different proportions.

### Bidirectional gated recurrent unit

In general, traditional recurrent neural networks (RNNs) can effectively encode the meaning of sentences as vector representation of words. However, RNNs often encounter issues with long-term dependencies. To resolve this, gated recurrent units (GRU) and long short-term memory (LSTM) networks were introduced, which enhance prediction results with fewer matrix multiplication operations^39–41^. Compared to LSTM, the structure of GRU is simpler and involves fewer parameters. Unlike LSTM, GRU incorporates an update gate ($\:{z}_{t}$) and a reset gate ($\:{r}_{t}$). At the current time step $\:t$, the $\:{z}_{t}$, $\:{r}_{t}$, and related internal calculation are defined as follows:

1 $$\:{z}_{t}=\:\sigma\:\:({U}_{z}{f}_{t}+\:{W}_{z}{h}_{t-1}+{b}_{z})$$

2 $$\:{r}_{t}=\sigma\:\:({U}_{r}{f}_{t}+\:{W}_{r}{h}_{t-1}+{b}_{r})$$

3 $$\:{c}_{t}=tanh\:({U}_{c}{f}_{t}+\:{r}_{t}({W}_{c}\odot{h}_{t-1})+{b}_{c})$$

4 $$\:{h}_{t}=\left(1-{z}_{t}\right)\odot{c}_{t}+{z}_{t}\odot{h}_{t-1}$$

where $\:{f}_{t}$ and $\:{h}_{t}$ represent the input and output at time step $\:t$, while $\:{h}_{t-1}$ represents the output from the previous time step $\:t-1$. $\:{c}_{t}$ denotes the candidate state. $\:{U}_{i}$, $\:{W}_{i}$, and $\:{b}_{i}$ represent the connection matrix, weight, and bias for $\:{z}_{t},$ $\:{r}_{t}$, and $\:{c}_{t}$, respectively.

Although the performance of GRU is better than that of RNN and LSTM, it cannot capture all key information due to its unidirectional propagation structure^41^. To address this limitation, bidirectional GRU (BiGRU), which consists of forward and backward neural networks, was developed to comprehensively capture information in both directions. At the current time step $\:t$, the time window size for the input is $\:d$. The inputs for the forward and backward GRU networks are $\:{f}_{t}(t=\text{1,2},\dots\:,d)$ and $\:{f}_{t}(t=d,\:d-1,\dots\:,1)$, respectively. In summary, the network structure of BiGRU is as follows:

5 $$\:{\overrightarrow{h}}_{t}=\:\overrightarrow{GRU}\:({\overrightarrow{h}}_{t-1},\:{f}_{t})(t=\text{1,2},\dots\:,d)$$

6 $$\:{\overleftarrow{h}}_{t}=\overleftarrow{GRU}({\overleftarrow{h}}_{t+1},\:{f}_{t})(t=t=d,\:d-1,\dots\:,1)$$

7 $$\:{h}_{t}=[{\overrightarrow{h}}_{t},{\overleftarrow{h}}_{t}]$$

### Self-attention mechanism

Attention is the process of selecting important information. To date, the attention mechanism has been successfully applied to several tasks in natural language processing (NLP), particularly in word embedding extraction^42,43^. Numerous studies have disclosed that the ATT approach significantly enhances model performance in bioinformatics and computational biology^44,45^. Herein, after obtaining the BiGRU-based feature embeddings, we employed the ATT mechanism to strengthen specific feature embeddings. The traditional ATT generates three standard matrices: Query (*Q*), Key (*K*) and Value (*V*). Thus, given an input, the attention calculation is defined as follows:

8 $$\:Attention\left(Q,K,V\right)=softmax\left(\frac{Q{K}^{T}}{\sqrt{d}}\right)$$.

where, $\:Q={W}_{q}X$, $\:K={W}_{k}X$, and $\:V={W}_{v}X$, and *X* represents the input. The parameter $\:d$ is the dimensionality of $\:Q$ and $\:K$, while $\:{W}_{q}$, $\:{W}_{k}$, and $\:{W}_{v}$ are the weight matrices used to compute $\:Q$, $\:K$, and $\:V$, respectively.

### The framework of BGATT-GA

**Fig. 1:**
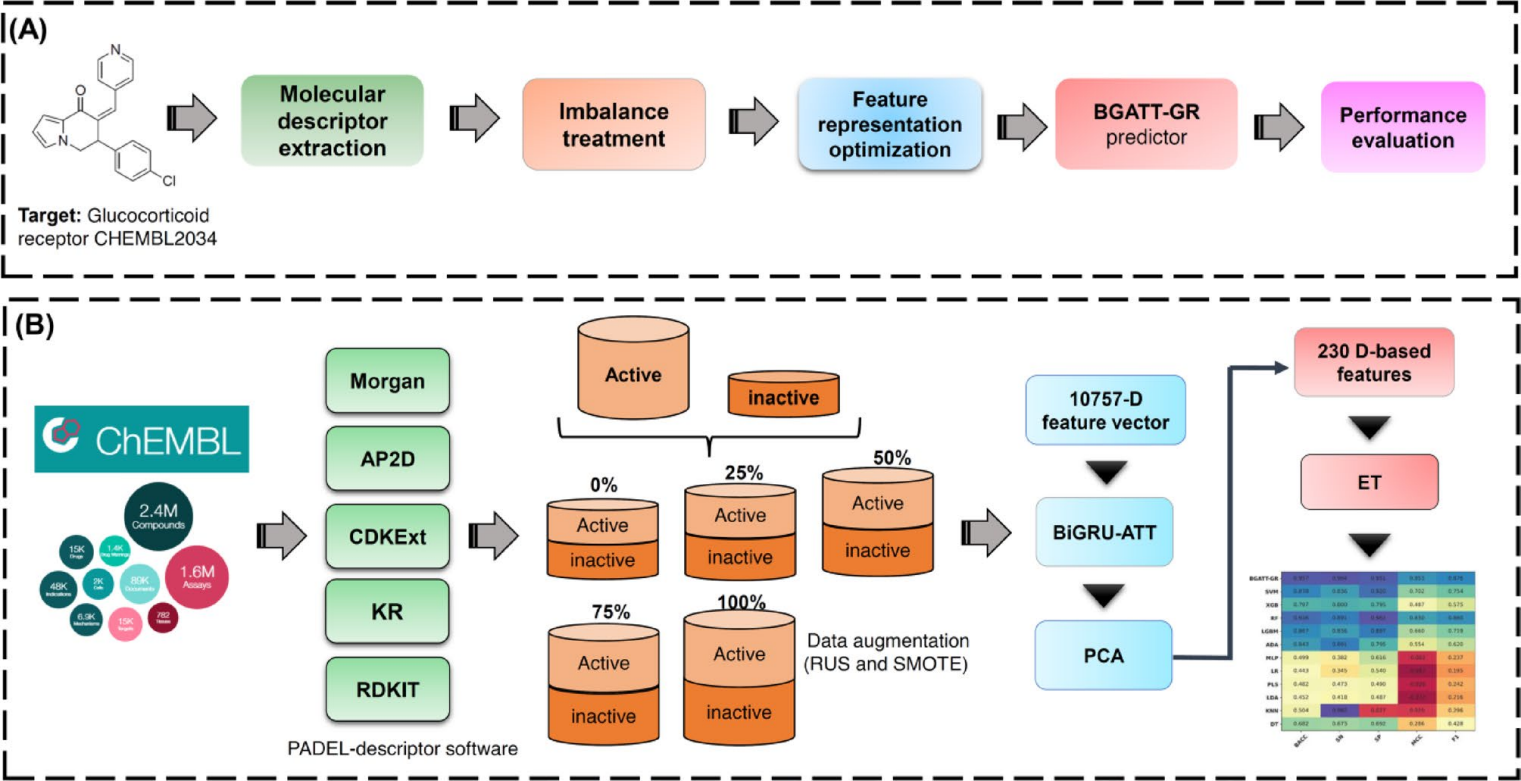
System flowchart of the proposed BGATT-GR. The overall workflow for the development of BGATT-GR contains five main steps: dataset preparation, molecular descriptor extraction, imbalance treatment, feature representation optimization, and performance evaluation.

The construction and performance evaluation process of BGATT-GR is summarized in Fig. 1. The source code and datasets are available at https://github.com/lawankorn-m/BGATT-GR. As shown in Fig. 1, there process comprises five main steps: dataset preparation, molecular descriptor extraction, imbalance treatment, feature representation optimization, and performance evaluation.

#### Step 1

Data preparation. The training and independent test datasets were obtained from small molecule compounds targeting the glucocorticoid receptor (Target ID: CHEMBL2034), which were sourced from the ChEMBL database.

#### Step 2

Molecular descriptor extraction. Five feature encoding methods were employed to encode the structural information of GR antagonists. These five feature representations were then combined to form a 10,757-D feature vector (termed Fusion).

#### Step 3

Imbalance treatment. Training the model on an extremely imbalanced dataset is ineffective. To attempt to resolve this, a data augmentation method combining both SMOTE and RUS techniques was employed to balance the dataset.

#### Step 4

Feature representation optimization. The dimensionality of Fusion feature vector was reduced using the BGATT architecture (Supplementary Figure** S1**) followed by principal component analysis (PCA). Since the 230 principal components could explained 95% of the data variance, the resulting feature vector has a reduced dimensionality of 230. Finally, the extremely randomized trees (ET) was used to develop the final model.

#### Step 5

Performance evaluation. The models developed in this study were evaluated using 10-fold cross-validation and independent tests. Performance metrics included BACC, MCC, AUPR, F1 score, area under the receiver operating characteristic (ROC) curve (AUC), sensitivity (SN), and specificity (SP)^37,38,44,46–51^. More information regarding these performance measures are provided in the Supplementary information.

## Results and discussion

### Performance assessment of different molecular descriptors

**Fig. 2:**
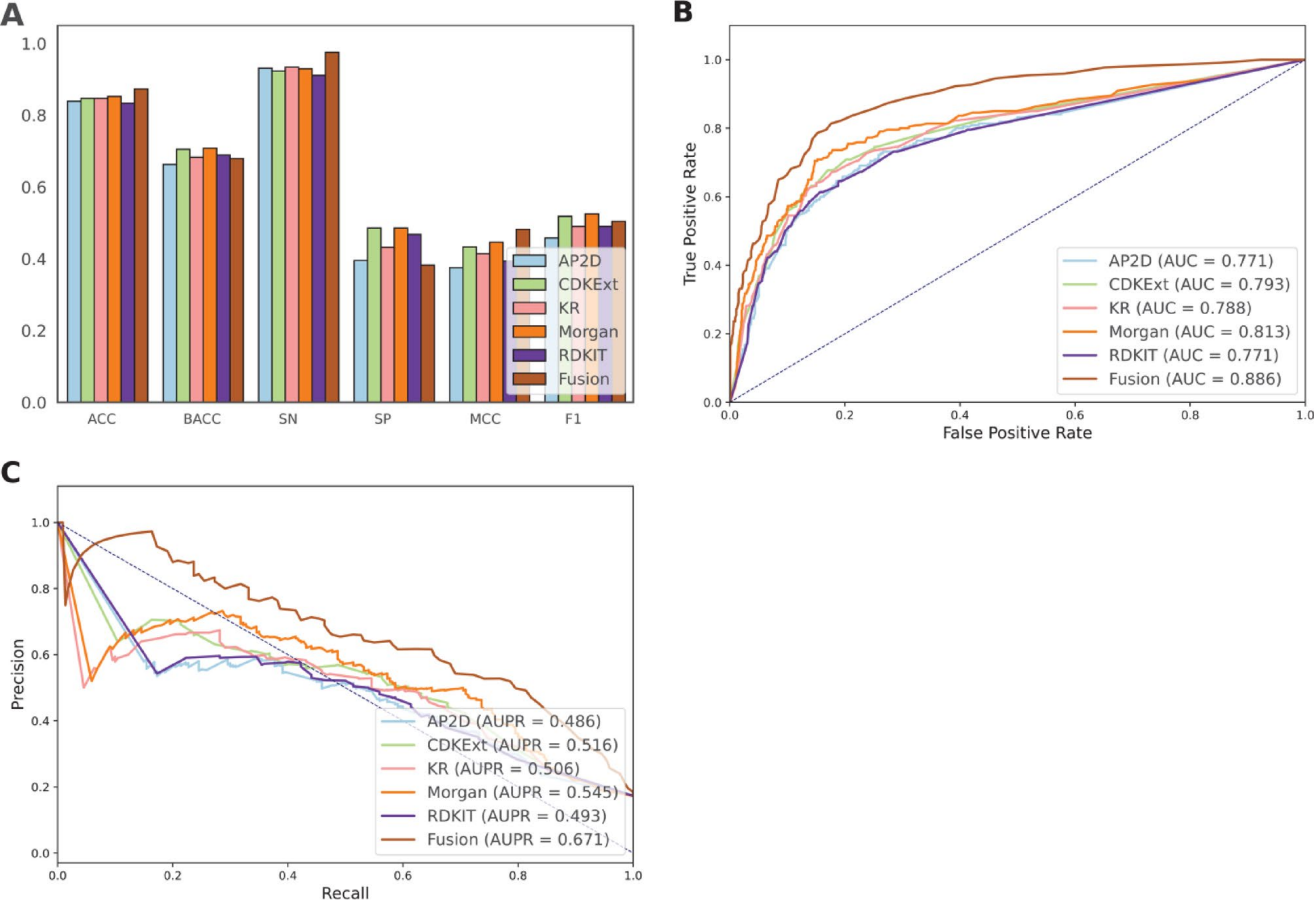
Performance comparison of different molecular on the training dataset. (**A**) Prediction results of different molecular descriptors in terms of ACC, BACC, SN, SP, MCC, and F1. (**B**) ROC curves and AUC values of different molecular descriptors. (**C**) PR curves and AUPR values of different molecular descriptors.

In this study, we employed various molecular descriptors and evaluated their effectiveness in identifying GR antagonists by training ET classifiers with five popular molecular descriptors, including AP2D, CDKExt, KR, Morgan, and RDKIT. Using these descriptors, we obtained feature vectors of dimensions 780-D, 1024-D, 4857-D, 2048-D, and 2048-D, respectively. In addition, to capture more comprehensive information about GR antagonists, we combined these descriptors to create a Fusion descriptor. The performance of the five individual-based molecular descriptors and Fusion in the cross-validation test is summarized in Fig. 2; Table 2. Furthermore, we plotted the ROC and PR curves to visually compare the performance of various molecular descriptors (Fig. 2B and C). As seen in Table 2, the AUPR scores of AP2D, CDKExt, KR, Morgan, RDKIT, and Fusion are 0.486, 0.516, 0.506, 0.545, 0.493, and 0.671, respectively. These results confirm that combining different molecular descriptors provides a more comprehensive view of GR antagonists, which significantly improves performance by capturing key information.

**Table 2:** Performance evaluation of various molecular descriptors over the cross-validation test on the training dataset.

Feature	BACC	SN	SP	MCC	AUC	F1	AUPR
AP2D	0.663	0.931	0.395	0.375	0.771	0.458	0.486
CDKExt	0.705	0.923	0.486	0.433	0.793	0.518	0.516
KR	0.683	0.934	0.432	0.414	0.788	0.490	0.506
Morgan	0.708	0.929	0.486	0.446	0.813	0.525	0.545
RDKIT	0.689	0.911	0.468	0.394	0.771	0.490	0.493
Fusion	0.679	0.975	0.382	0.482	0.886	0.504	0.671

### Analysis of applicability domain

To evaluate the reliability of predictions for new compounds, the applicability domain (AD) measures how closely these compounds resemble those used to train the model^19,22,29,30,34,52^. By applying the t-Distributed Stochastic Neighbor Embedding (t-SNE) technique^53^, the distribution of training and independent datasets was visualized, as shown in Supplementary Figure S2. The t-SNE method constructs a probability-based mapping that reflects the degree of similarity between data points within a complex, high-dimensional space. Analysis based on molecular descriptors revealed that the vast majority of compounds in the independent test dataset cluster together with those from the training dataset. This clustering strongly indicates that the chosen molecular descriptors are optimal for building reliable prediction models.

### Data augmentation method is able to solve the data imbalance problem

**Fig. 3:**
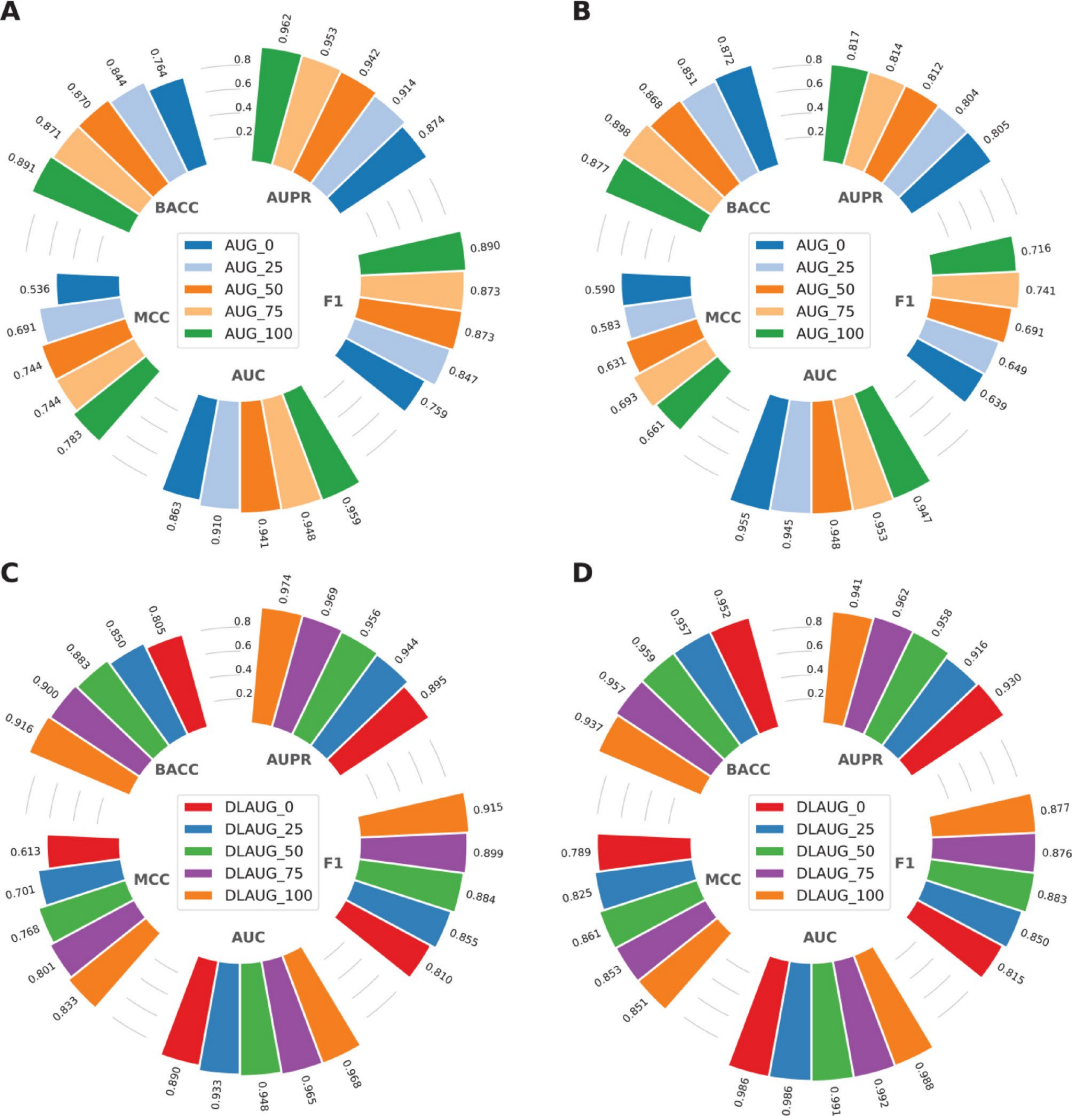
Performance comparison of different proportions on the training (**A**,** C**) and independent test (**B**,** D**) datasets. **(A–B)** Prediction results of the data augmentation method with different proportions in terms of BACC, MCC, F1, AUC, and AUPR. **(C–D)** Prediction results of the BiGRU-ATT architecture coupled with the data augmentation method.

Although Fusion outperformed the other molecular descriptors, its predictive ability in identifying GR antagonists remains unsatisfactory. For instance, its SP score was only 0.382. The extreme imbalance between active and inactive samples (i.e., 1054 actives and 214 inactives) was a major contributing factor to the low overall performance. To resolve this issue, we employed an effective data augmentation method that combines both the SMOTE and RUS techniques. In brief, the SMOTE method was used to oversample the inactive samples at various proportions, including 0%, 25%, 50%, 75%, and 100%. Simultaneously, an equal number of active samples were randomly selected from the positive dataset to construct balanced datasets, referred to as AUG_0, AUG_25, AUG_50, AUG_75, and AUG_100, respectively. The performance of the models trained using these balanced datasets is recorded in Fig. 3A and B; Table 3. We noticed that the performance of the models trained on all five balanced datasets was significantly higher than that of the models trained on the original, highly imbalanced dataset. Specifically, the AUPR scores for the balanced datasets ranged from 0.871 to 0.962 in the cross-validation test, compared to an AUPR score of 0.671 for the original dataset. Moreover, models trained on the balanced datasets significantly outperformed the control model in terms of BACC, MCC, and F1 score, underscoring the effectiveness of the data augmentation method in resolving the imbalance issue. Thus, we utilized this data augmentation method to develop the proposed model in the forthcoming studies.

**Table 3:** Performance evaluation of the data augmentation method with different proportions over the cross-validation and independent tests on the training and independent test datasets, respectively.

Evaluation strategy	Proportion	BACC	SN	SP	MCC	AUC	F1	AUPR
Cross-validation	AUG_0	0.764	0.741	0.786	0.536	0.863	0.759	0.874
	AUG_25	0.844	0.859	0.828	0.691	0.910	0.847	0.914
	AUG_50	0.870	0.888	0.852	0.744	0.941	0.873	0.942
	AUG_75	0.871	0.879	0.862	0.744	0.948	0.873	0.953
	AUG_100	0.891	0.877	0.905	0.783	0.959	0.890	0.962
Independent test	AUG_0	0.872	0.964	0.779	0.590	0.955	0.639	0.805
	AUG_25	0.851	0.873	0.829	0.583	0.945	0.649	0.804
	AUG_50	0.868	0.873	0.863	0.631	0.948	0.691	0.812
	AUG_75	0.898	0.909	0.886	0.693	0.953	0.741	0.814
	AUG_100	0.877	0.873	0.882	0.661	0.947	0.716	0.817

### The proposed BGATT contributes to performance improvement

Although the data augmentation method improved performance in identifying GR antagonists, its effectiveness on the independent test remained limited due to the high dimensionality of Fusion. to address this, the BGATT architecture was introduced to reduce the dimensionality of Fusion. The dimensionality reduction process using BGATT is briefly described as follows. First, each balanced dataset was fed into the BGATT architecture to generate a feature embedding with a dimensionality of 4000. Second, the redundant information in the feature embedding was removed using the PCA approach. Third, the PCA-transformed feature vector was then fed into ET classifiers along with different balanced training datasets processed by the data augmentation method. For the ease of discussion, the PCA-transformed feature representations corresponding to different data augmentation proportions (i.e., 0%, 25%, 50%, 75%, and 100%) are referred to as DLAUG_0, DLAUG _25, DLAUG_50, DLAUG_75, and DLAUG_100, respectively. The detailed prediction results for these feature representations under both evaluation strategies are presented in Fig. 3C and D; Table 4. The AUPR scores for DLAUG_0, DLAUG _25, DLAUG_50, DLAUG_75, and DLAUG_100 during the cross-validation test were 0.895, 0.944, 0.956, 0.969, and 0.974, respectively. For the independent test, the AUPR scores were 0.930, 0.916, 0.958, 0.962, and 0.941, respectively. Considering the results of both evaluation strategies, DLAUG_75 and DLAUG_100 exhibited similar performance and outperformed other feature representations. Specifically, the BACC, SN, MCC, AUC, and AUPR for DLAUG_75 were 0.957, 0.964, 0.853, 0.992, and 0.962, respectively, representing improvements of 1.97, 5.45, 0.14, 0.42, and 2.06% over DLAUG_100. Altogether, these results indicate that DLAUG_75 achieved superior and more stable performance. Therefore, we utilized DLAUG_75 to optimize the proposed BGATT-GR model.

**Table 4:** Performance evaluation of the BGATT architecture coupled with the data augmentation method over the cross-validation and independent tests on the training and independent test datasets, respectively.

Evaluation strategy	Proportion	BACC	SN	SP	MCC	AUC	F1	AUPR
Cross-validation	DLAUG_0	0.805	0.832	0.777	0.613	0.890	0.810	0.895
	DLAUG_25	0.850	0.867	0.833	0.701	0.933	0.855	0.944
	DLAUG_50	0.883	0.888	0.879	0.768	0.948	0.884	0.956
	DLAUG_75	0.900	0.888	0.912	0.801	0.965	0.899	0.969
	DLAUG_100	0.916	0.900	0.932	0.833	0.968	0.915	0.974
Independent test	DLAUG_0	0.952	1.000	0.905	0.789	0.986	0.815	0.930
	DLAUG_25	0.957	0.982	0.932	0.825	0.986	0.850	0.916
	DLAUG_50	0.959	0.964	0.954	0.861	0.991	0.883	0.958
	DLAUG_75	0.957	0.964	0.951	0.853	0.992	0.876	0.962
	DLAUG_100	0.937	0.909	0.966	0.851	0.988	0.877	0.941

### Selection of the optimal classifier

**Fig. 4:**
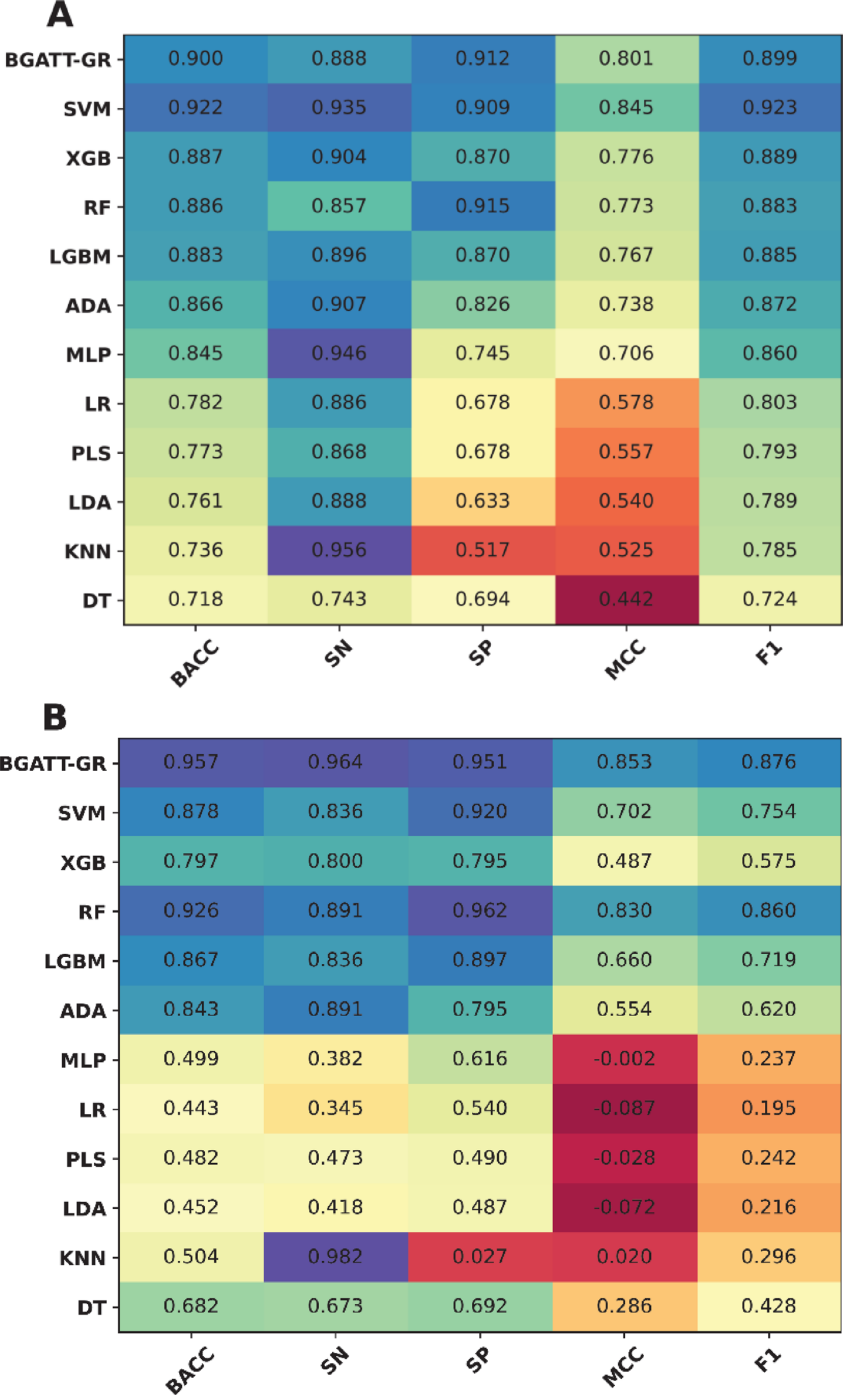
Heat-map of the prediction results of BGATT-GR and conventional ML classifiers on the training (**A**) and independent test (**B**) datasets.

**Fig. 5:**
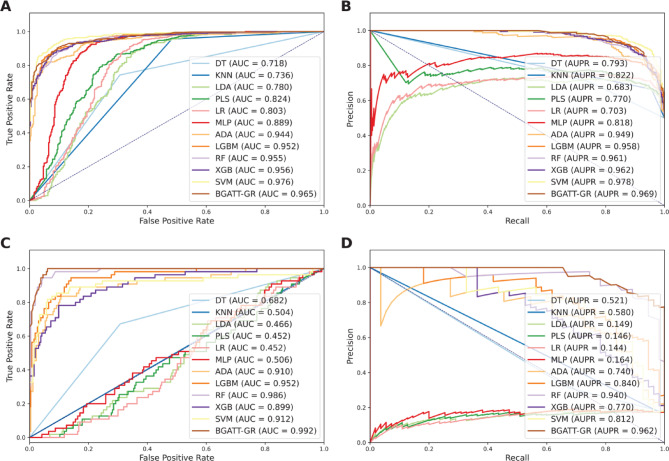
Performance comparison of BGATT-GR and conventional ML classifiers. Comparisons of the ROC curve, AUC value, PR curve, and AUPR value on the training (**A**,** B**) and independent test (**C**,** D**) datasets.

To evaluate the effectiveness of BGATT-GA in identifying GR antagonists, we compared its performance with eleven popular ML classifiers, i.e., MLP, ADA, RF, SVM, XGB, light gradient boosting machine (LGBM), decision tree (DT), k-nearest neighbor (KNN), linear discriminant analysis (LDA), partial least squares (PLS), and logistic regression (LR). To date, these classifiers have been widely used to biological and chemical classification tasks^29,52,54–57^. The optimization and construction procedures for these eleven classifiers follow the same approach used in our previous studies, with their optimal parameters determined through a grid search strategy combined with 10-fold cross-validation, as shown in Supplementary Table 2^29,30,52,57–59^. Figure 4; Table 5 summarize the performance evaluation of BGATT-GR and the conventional ML classifiers in both cross-validation and independent tests. The independent test was conducted to assess whether the model suffers from overfitting. In addition, we plotted the ROC and PR curves to intuitively compare the performance of BGATT-GR and the conventional ML classifiers (Fig. 5). From Fig. 4; Table 5, SVM demonstrates the best performance among the ML classifiers in the cross-validation test, with an AUPR score of 0.978. BGATT-GR and XGB attained the second-best and third-best performances, with AUPR scores of 0.969 and 0.962, respectively. However, in the independent test, BGATT-GR outperformed SVM and all other ML classifiers, achieving an AUPR score of 0.962, compared to 0.812 for SVM and 0.770 for XGB. Furthermore, the AUPR, BACC, SN, and MCC of BGATT-GR were 15.03–19.19, 7.88–15.98, 12.73–16.36, and 15.10–36.55% higher, respectively, than those of SVM and XGB. Taken together, these results highlight that BGATT-GR exhibits stable performance, strong robustness, and excellent generalization ability.

**Table 5:** Performance evaluation and comparison of BGATT-GR and conventional ML classifiers over the cross-validation and independent tests on the training and independent test datasets, respectively.

Evaluation strategy	Method	BACC	SN	SP	MCC	AUC	F1	AUPR
Cross-validation	DT	0.718	0.743	0.694	0.442	0.718	0.724	0.793
	KNN	0.736	0.956	0.517	0.525	0.736	0.785	0.822
	LDA	0.761	0.888	0.633	0.540	0.780	0.789	0.683
	PLS	0.773	0.868	0.678	0.557	0.824	0.793	0.770
	LR	0.782	0.886	0.678	0.578	0.803	0.803	0.703
	MLP	0.845	0.946	0.745	0.706	0.889	0.860	0.818
	ADA	0.866	0.907	0.826	0.738	0.944	0.872	0.949
	LGBM	0.883	0.896	0.870	0.767	0.952	0.885	0.958
	RF	0.886	0.857	0.915	0.773	0.955	0.883	0.961
	XGB	0.887	0.904	0.870	0.776	0.956	0.889	0.962
	SVM	0.922	0.935	0.909	0.845	0.976	0.923	0.978
	BGATT-GR	0.900	0.888	0.912	0.801	0.965	0.899	0.969
Independent test	DT	0.682	0.673	0.692	0.286	0.682	0.428	0.521
	KNN	0.504	0.982	0.027	0.020	0.504	0.296	0.580
	LDA	0.452	0.418	0.487	−0.072	0.466	0.216	0.149
	PLS	0.482	0.473	0.490	−0.028	0.452	0.242	0.146
	LR	0.443	0.345	0.540	−0.087	0.452	0.195	0.144
	MLP	0.499	0.382	0.616	−0.002	0.506	0.237	0.164
	ADA	0.843	0.891	0.795	0.554	0.910	0.620	0.740
	LGBM	0.867	0.836	0.897	0.660	0.952	0.719	0.840
	RF	0.926	0.891	0.962	0.830	0.986	0.860	0.940
	XGB	0.797	0.800	0.795	0.487	0.899	0.575	0.770
	SVM	0.878	0.836	0.920	0.702	0.912	0.754	0.812
	BGATT-GR	0.957	0.964	0.951	0.853	0.992	0.876	0.962

### Visualization of feature representations

**Fig. 6:**
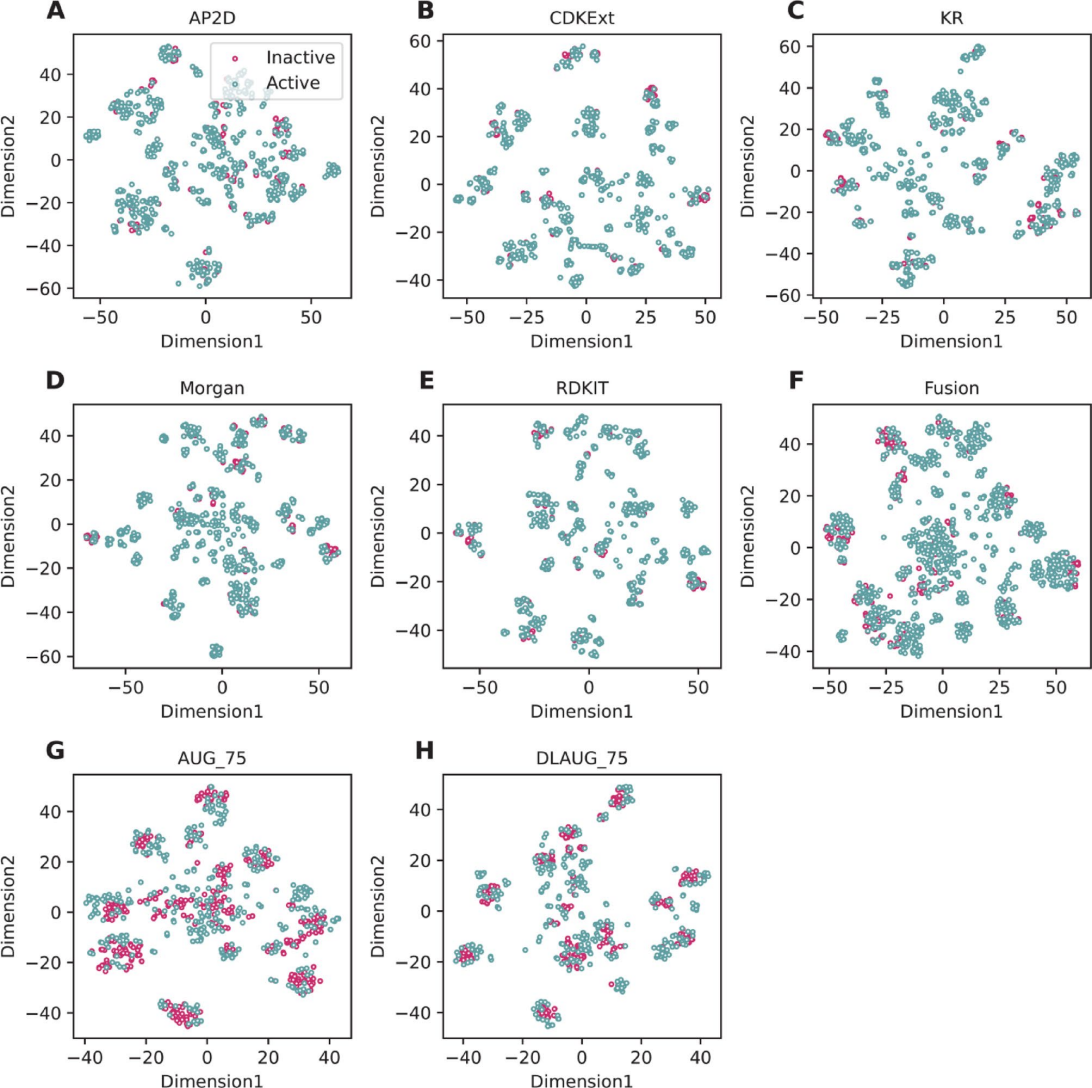
Comparison of t-SNE plots of different feature representations on the training dataset, where green and red dots represent active and inactive compounds, respectively.

BGATT-GR, developed based on DLAUG_75, demonstrated better and more stable performance in both cross-validation and independent test results. To intuitively illustrate the reason for this, we applied the t-distributed Stochastic Neighbor Embedding (t-SNE) approach^53^ to various feature representations, including the five molecular descriptors (AP2D, CDKExt, KR, Morgan, and RDKIT), Fusion, AUG_75, and DLAUG_75. Here, t-SNE was used to map the high-dimensional feature vectors into a two-dimensional feature space to compare the feature representation ability of different features. In the visualizations, green and red dots represent active and inactive compounds, respectively. As seen in Fig. 6A and F, the green and red dots corresponding to the five molecular descriptors and Fusion almost completely overlap, indicating limited ability to distinguish active compounds from inactive ones. In contrast, when AUG_75 and DLAUG_75 were used, the separation between green and red dots became more distinct (Fig. 6G and H). These results confirm that the proposed feature representation (DLAUG_75) can provide more discriminative power for identifying GR antagonists.

### Ablation experiments

To understand how our DL-based framework, which combines the data augmentation method and the BGATT architecture, intrinsically influences BGATT-GR, we investigated the contribution of these components to the identification of GR antagonists. To achieve this, we conducted ablation experiments by excluding these two components from the proposed model. We defined BGATT-GR trained with different combinations of these components as follows:

(i) (−) AUG + (−) BGATT: Exclude both the data augmentation method and the BGATT architecture (baseline strategy).
(ii) (+) AUG + (−) BGATT: Include the data augmentation method but excludes the BGATT architecture.
(iii) (−) AUG + (+) BGATT: Include the BGATT architecture but excludes the data augmentation method.
(iv) (+) AUG + (+) BGATT: Include both the data augmentation method and the BGATT architecture (complete model).

**Fig. 7:**
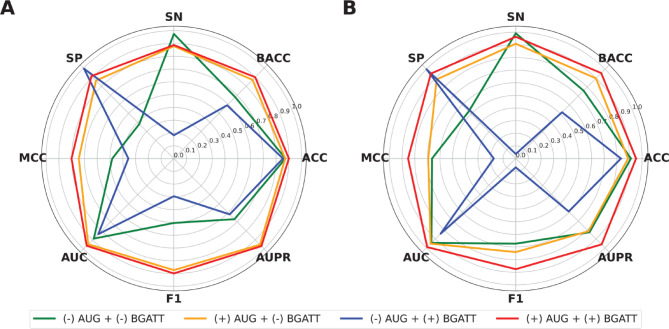
The prediction results of ablation experiments on the training (**A**) and independent test (**B**) datasets, where Strategies 1–4 represent BGATT-GR based on (−) AUG + (-) BGATT, (+) AUG + (−) BGATT, (−) AUG + (+) BGATT, and (+) AUG + (+) BGATT, respectively.

The prediction results of the ablation experiments for the training and independent test datasets are summarized in Fig. 7; Table 6. The results show that excluding the BGATT architecture and the data augmentation method caused reductions in AUPR of 29.82, 1.65, and 35.08%, respectively, during the cross-validation test. For the independent test, these exclusions led to AUPR reductions of 13.65, 14.76, and 36.88%, respectively. These findings highlight the critical role of both the data augmentation method and the BGATT architecture in enabling BGATT-GR to attain excellent predictive capacity and generalization ability.

**Table 6:** The results of ablation experiments over the cross-validation and independent tests on the training and independent test datasets, respectively.

Evaluation strategy	Strategy	BACC	SN	SP	MCC	AUC	F1	AUPR
Cross-validation	(−)AUG + (−) BGATT	0.679	0.975	0.382	0.482	0.886	0.504	0.671
	(+)AUG + (−) BGATT	0.871	0.879	0.862	0.744	0.948	0.873	0.953
	(-)AUG + (+) BGATT	0.589	0.182	0.995	0.356	0.837	0.295	0.618
	(+)AUG + (+) BGATT	0.900	0.888	0.912	0.801	0.965	0.899	0.969
Independent test	(−)AUG + (−) BGATT	0.760	0.992	0.527	0.663	0.945	0.674	0.826
	(+)AUG + (−) BGATT	0.898	0.909	0.886	0.693	0.953	0.741	0.814
	(−)AUG + (+) BGATT	0.518	0.036	1.000	0.174	0.842	0.070	0.593
	(+)AUG + (+) BGATT	0.957	0.964	0.951	0.853	0.992	0.876	0.962

## Conclusions

The accurate and efficient identification of GR antagonists is crucial for both basic research and drug development. In this study, we propose a new deep learning-based hybrid framework, named BGATT-GR, for identifying GR antagonists. This framework maximizes the utility of multi-view features, the data augmentation method, the BGATT architecture, and PCA. Both cross-validation and independent test results confirm that BGATT-GR demonstrates robustness and excellent generalization ability on imbalanced datasets, with AUPR scores of 0.969 and 0.962, respectively. When compared with conventional ML classifiers on the independent test dataset, BGATT-GR significantly outperformed these models, with an MCC of 0.853, BACC of 0.957, and an AUC of 0.992. The excellent performance of BGATT-GR can be attributed to the following factors: (i) combining both RUS and SMOTE effectively addresses the class imbalance issue; (ii) the generation of multi-view features captures critical information about GR antagonists; and (iii) the construction of BGATT architecture enhances the discriminative power of the multi-view features for the identification of GR antagonists. We anticipate that BGATT-GR will serve as a valuable tool for accelerating the discovery of novel GR antagonists in a cost-effective manner. Furthermore, the hybrid framework and data augmentation method developed in this study can be applied to address various research challenges in other chemical classification problems.

## Electronic supplementary material

Below is the link to the electronic supplementary material.

Supplementary Material 1

## Data Availability

The datasets used in this study can be downloaded through the following link: https://github.com/lawankorn-m/BGATT-GR.
