# Supplementary material for: BGATT-GR: accurate identification of glucocorticoid receptor antagonists based on data augmentation combined with BiGRU-attention
Source: Sci Rep. 2025 Jul 1;15:21402. doi: 10.1038/s41598-025-05839-8 (PMC12218296; doi:10.1038/s41598-025-05839-8)
Supplement: Supplementary file 1 — Supplementary Material 1 [file 41598_2025_5839_MOESM1_ESM.docx]

### **Performance evaluation**

Herein, SN, BACC, MCC, AUC, AUPR, F1, and SP were adopt to evaluate the predictive ability and robustness of the models. These performance measures are defined by the following equations:

| $SN=\frac{\mathrm{TP}}{\left( TP+FN \right)}$ | (1) |
| --- | --- |
| $SP=\frac{\mathrm{TN}}{\left( TN+FP \right)}$ | (2) |
| $ACC=\frac{TP+TN}{\left( TP+TN+FP+FN \right)}$ | (3) |
| $BACC=(SN+SP)\times0.5$ | (4) |
| $MCC=\frac{TP\times TN-FP\times FN}{\sqrt{(TP+FP)(TP+FN)(TN+FP)(TN+FN)}}$ | (5) |
| $F1=2\times\frac{\mathrm{TP}}{2TP+FP+FN}$ | (6) |

where the numbers of correctly predicted positive and negative samples were referred to as TP and TN, respectively. Conversely, the numbers of falsely predicted positive and negative samples are referred to as FP and FN, respectively.


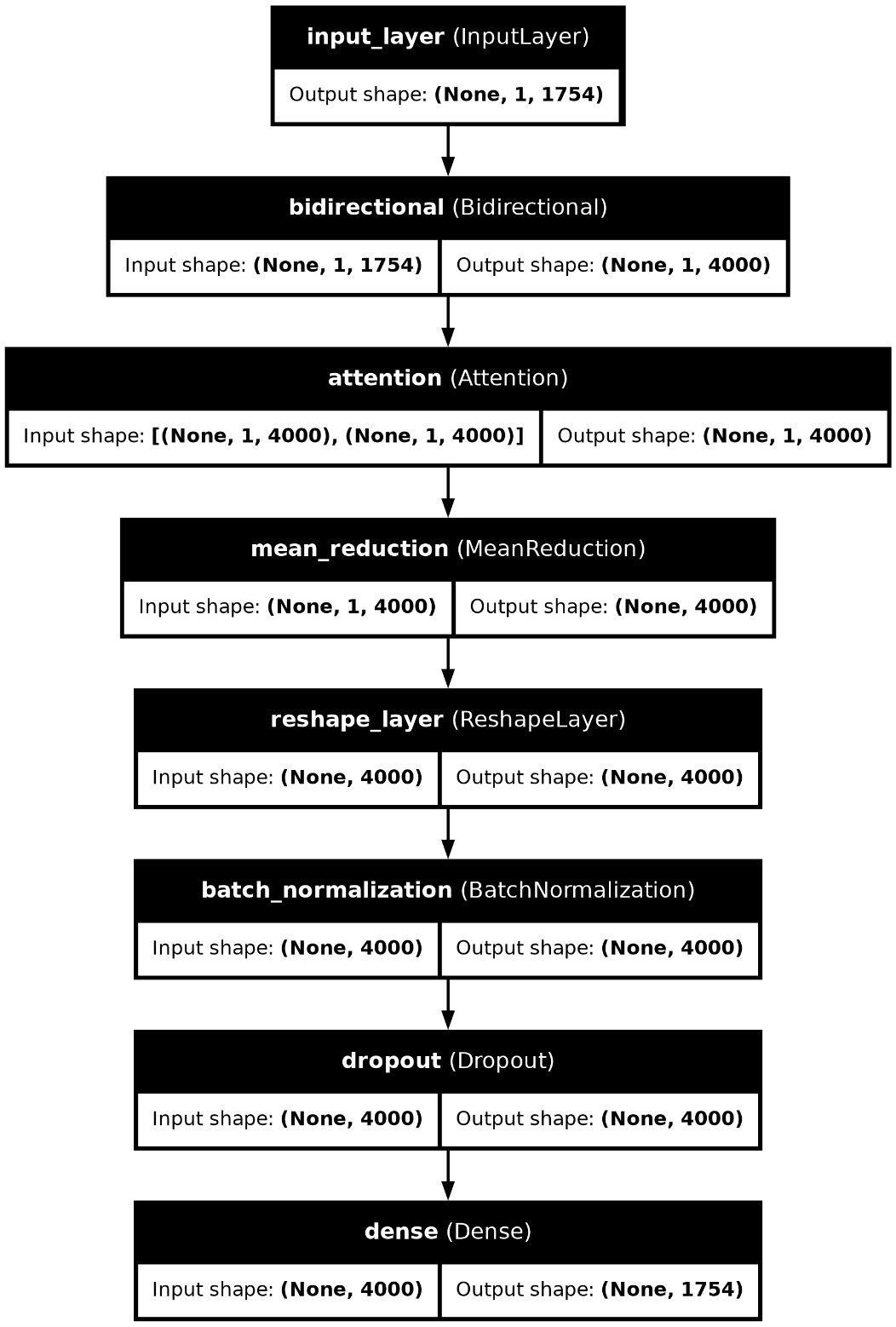


## Figure S1 Overall architecture for BGATT-GR

## Figure S2 Analysis of applicability domain of AP2D, CDKExt, KR, Morgan, and RDKIT by t-distributed stochastic neighbor embedding (t-SNE)

## Table S1. Summary of training and independent test datasets at different proportions

| **Proportion (%)** | **Training dataset** | | **Independent test dataset** | |
| --- | --- | --- | --- | --- |
|  | **Active** | **Inactive** | **Active** | **Inactive** |
| 0 | 220 | 220 | 214 | 61 |
| 25 | 275 | 275 | 214 | 61 |
| 50 | 330 | 330 | 214 | 61 |
| 75 | 385 | 385 | 214 | 61 |
| 100 | 440 | 440 | 214 | 61 |

## Table S2. Information of parameter settings for twelve ML methods used in this study.

| **Method** | **Parameter** | **Search space** |
| --- | --- | --- |
| ADA | n_estimators | [20, 50, 100, 200, 500] |
| DT | max_depth | 2–20 with an interval of 1. |
| ET | n_estimators | [20, 50, 100, 200, 500] |
| KNN | number of neighbours | 1–150 with an interval of 1 |
| LGBM | n_estimators | [20, 50, 100, 200, 500] |
| LR | C | np.logspace(-3, 3, num=100) |
| MLP | hidden_layer_sizes | [20, 50, 100, 200, 500] |
| NB | var_smoothing | np.logspace(0,-9, num=100) |
| PLS | #Components | 10–1000 with an interval of 10 |
| RF | n_estimators | [20, 50, 100, 200, 500] |
| SVM | Cost | [2^-4^–2^4^] in log_2_ steps |
| XGB | n_estimators | [20, 50, 100, 200, 500] |

Columns 2 and 3 represents the parameter name used in the Scikit-learn library and the range of parameter used to develop the model, respectively.
